# Visual Monitoring Strategies of Sentinels in a Cooperative Breeder

**DOI:** 10.3390/biology11121769

**Published:** 2022-12-06

**Authors:** Guy Beauchamp, Reed Bowman

**Affiliations:** 1 Independent Researcher, Montreal, QC, Canada; 2 Archbold Biological Station, 123 Main Dr., Venus, FL 33960, USA

**Keywords:** bird, group size, log-normal distribution, predation, sentinel behaviour, vigilance

## Abstract

**Simple Summary:**

Animals frequently interrupt their activities to monitor their surroundings for possible threats such as predators and intruders. How animals carry out this vigilance has received little attention. In particular, the quality of vigilance depends on where animals look and how long each look lasts. We examine how vigilance is organized in the Florida scrub-jay (*Aphelocoma caerulescens*). During vigilance, these birds turn their heads in different directions to detect threats. We found that birds turned their heads regularly and also regularly returned their gaze to areas previously monitored, which is predicted when predators and intruders rely on surprise to approach. Birds turned their heads in several directions during vigilance, but often more frequently on one side of the body than the other, which was not predicted for regular vigilance. Looks were shorter in smaller groups and in juveniles presumably to increase visual coverage in more threatening situations. Visual monitoring strategies during vigilance reflect the risk posed by predators and intruders.

**Abstract:**

Vigilance is important for early detection of threats. Previous studies have focused on the allocation of time to vigilance but neglected how animals monitor their surroundings during vigilance. Where animals look and how long each look lasts can affect the quality of visual monitoring and thus the ability to detect threats during vigilance. We examined visual monitoring strategies in the Florida scrub-jay (*Aphelocoma coerulescens*), a cooperative breeder with sentinel behaviour. Sentinels in this species make head turns from vantage points to detect the arrival of predators and intruding neighbours. We found that sentinels initiated head turns at regular intervals and also returned their gaze to areas previously monitored at regular intervals, which is predicted when predators and intruders rely on surprise rather than stealth to approach. Sentinels made head turns in several directions, but often more frequently on one side of the body than the other, which was not predicted for regular vigilance. Average look duration during sentinel bouts was shorter in smaller groups and in juveniles. We argue that shorter looks are beneficial to increase visual coverage in more threatening situations. Our study highlights how visual monitoring strategies during vigilance reflect the risk posed by predators and intruders.

## 1. Introduction

Threats from predators or rivals are ever present in the lives of many animals. Early detection of these threats is key to reduce fitness losses. Early detection can be achieved by diverting time from other activities to vigilance [1]. Using various senses, vigilant animals can monitor their surroundings for potential danger, thus increasing their chances of detecting threats before it is too late to respond. Increasing time spent vigilant, when possible, should increase the chances of detecting threats early [2,3,4].

In addition to the overall time spent vigilant, the quality of monitoring during vigilance can also influence the ability to detect threats [5]. To increase the quality of monitoring, animals could focus more attention on vigilance during their activities or coordinate vigilance with neighbours. How animals actually monitor their surroundings during vigilance could also matter. For instance, an animal that only monitors one area during vigilance would have little chances of detecting threats coming from other directions regardless of the time allocated to vigilance. Given that monitoring in all directions is not possible at any given time for most species, animals must use strategies to monitor their surroundings in a way that increases the ability to detect a variety of threats. Some animals, for example, turn their heads during vigilance bouts to visually explore different areas in their surroundings [6]. The frequency of head turns influences where animals look at any given time and how long each look lasts, two key features to increase visual coverage during vigilance and thus the chances of detecting threats early [7].

Empirical research on visual monitoring strategies during vigilance is limited. Some studies have documented head turns but calculated their frequency over bouts of foraging and vigilance thus weakening a possible association with threat detection [8,9]. Other studies have reported an increase in the frequency of head turns when predation risk is high [5,7,10], which supports the idea that head turns are associated with threat detection. The greatest challenge thus far in studies of visual monitoring strategies is uncertainty about the targets of vigilance. For group foragers, for instance, vigilance may be aimed at distant predators or at nearby rivals [11,12]. Focusing on one type of threat might affect the ability to detect the other, and so distinct strategies to monitor each threat might be needed. In addition, individuals might be looking for foraging opportunities nearby rather than monitoring distant threats [13,14,15]. Because visual monitoring strategies might be tailored to specific targets of vigilance, it is important to establish the targets of vigilance. While it might be possible to infer targets of vigilance from gaze direction, this is problematic for species such as birds with eyes set laterally rather than frontally with broad visual coverage [16].

Sentinel behaviour is an ideal system to examine how animals visually monitor their surroundings during vigilance. During sentinel behaviour, individuals monitor their surroundings from vantage points and coordinate their vigilance with one another at the group level [17,18]. While not common in animals, sentinel behaviour is known in species of fish, birds, and mammals. Sentinels are not seeking resources and are not competing with one another to become or remain a sentinel. Indeed, sentinel bouts can be initiated or terminated in response to hunger levels [19]. Sentinels can thus direct most of their attention to distant threats, such as predators or intruders, rather than nearby foraging opportunities or other group members foraging below.

In this study, we examined visual monitoring strategies in the Florida scrub-jay (*Aphelocoma coerulescens*), a cooperative breeder with sentinel behaviour. Families of Florida scrub-jays live in large, all-purpose territories year-round [20]. Sentinel behaviour occurs frequently in this species and is performed by all group members albeit more often by breeders than helpers or juveniles [21]. Sentinel behaviour peaks in the non-breeding season, corresponding with the peak in abundance of aerial predators [21]. Previous studies with this species have focused on the amount of time allocated to sentinel behaviour [22,23,24], but not on visual monitoring strategies that sentinels can use for threat detection. In the non-breeding season, sentinels monitor their surroundings for aerial predators such as Cooper’s hawks (*Accipiter cooperi*) and falcons (*Falco* spp.) [25], which rely on surprise rather than stealth to approach their prey. Sentinels also monitor neighbours from abutting territories for possible intrusions. Disputes with neighbours are about maintaining territorial boundaries rather than preventing surreptitious thefts of limited resources [20].

Theory suggests that animals should adopt regular, systematic vigilance for early detection of threats that can emerge from almost any direction at any time [26,27] such as aerial predators and intruders in Florida scrub-jays. Looks of various duration, for instance, would be dangerous because short looks might be insufficient to detect threats and longer looks in one direction would limit the ability to detect threats coming from other directions. In light of the theory, we predicted that Florida scrub-jay sentinels should initiate head turns at regular intervals to monitor different areas and return their gaze to areas previously monitored at regular intervals. Birds such as Florida scrub-jays have eyes set laterally rather than frontally [28]. These birds have a small binocular area in front of them, a rather large blind area to the back of the head, and two broad monocular areas on either side. Their eyes have limited movements so that gaze direction is mostly governed by head turns. To achieve broad, systematic monitoring, head turns in sentinels should occur as frequently on each side of the body and have the same amplitude. Such head turns would allow sentinels to monitor all areas systematically including the blind area behind their heads [28]. Regular visual monitoring need not imply that average look duration is the same for all individuals. Florida scrub-jay families vary in size and may include juveniles as well as adults of different ages. If look duration is adapted to the level of risk experienced by different individuals, then differences in look duration might be expected among different classes of birds and among families.

## 2. Materials and Methods

The study took place at Archbold Biological Research Station in south-central Florida (USA). The area is characterized by scrub oaks (*Quercus* spp.) rarely more than 2 m tall. The station population has been ringed for decades now and is monitored regularly to evaluate demographic variables [20]. At the time of the study, sex was known for helpers and breeders but not for juveniles. More than 80 territories were mapped and could be accessed by using a network of trails. We carried out observations from late February to early March 2022, which corresponds to the onset of the breeding season. At this time of year, juveniles from the previous breeding season are close to one year-old.

### 2.1. Sampling

Each day, one of us walked a different series of trails to encounter as many different groups as possible. Birds were observed from 7:00 to 11:00 in the morning with one exception one afternoon. Typically, about five groups were monitored daily ranging from four to seven. During walks, the observer attempted to locate foraging groups performing sentinel behaviour. Sentinel behaviour is easily distinguished and occurs frequently during the day in groups of all sizes [21,22]. Sentinels occupy a perch on top of a bush or in a small tree from which they scan the horizon with frequent head turns. Once sentinel behaviour in a group was detected, the observer used a video camera to record the behaviour of one sentinel bird at a time. Florida scrub-jays at the station are accustomed to human presence, which allows observations to be made at close range without obvious disturbances. With the help of an 80× zoom on the video camera, the observer could get close-ups that easily allowed detection of head movements. Observations lasted for a scheduled 6 min unless the bird left the sentinel perch or changed body position. No changes in body position means that the area monitored by a sentinel after a head turn in a particular direction remains the same. No more than three observations were carried out with a given group on a given day. In addition to the video recordings, the observer also noted the number of birds present in the immediate vicinity of the focal bird to get an estimate of group size (typically the whole family is present). Identity and social status (juvenile, helper, male or female breeder) were obtained later using information from the coloured rings.

Sentinel scrub-jays utter alarm calls when detecting aerial predators [25]. Such alarms calls were not detected during our focal observations. Stereotypical displays and calling accompany detection of nearby intruders [25]. We did not collect data when such interactions between groups occurred at territory boundaries as sentinel behaviour was rarely performed.

### 2.2. Video Analysis

Head movements during sentinel behaviour were timed using frame-by-frame analysis of video recordings. For each video, the times at which each detectable head movement started and finished was recorded. This allowed us to get the duration of two intervals: the duration of the interval during which the head moves prior to a look (head move duration) and the duration of the look prior to the next head move (look duration). The minimum time resolution was 0.033 s due to the video frame rate. Any head moves or looks lasting less than this limit could not be detected, but this appears unlikely given that head moves typically lasted 2 or 3 time frames and looks were much longer (see Section 3). As the duration of head moves was short and close to the limit of detection, we focused on the distribution of looks only. Notice that the duration of a look excludes the duration of the head move preceding it. This is justifiable because vision is blurred during head moves [29]. In addition, head moves are often associated with eye blinks [30,31], which would reduce the ability to detect threats.

**Figure 1:**
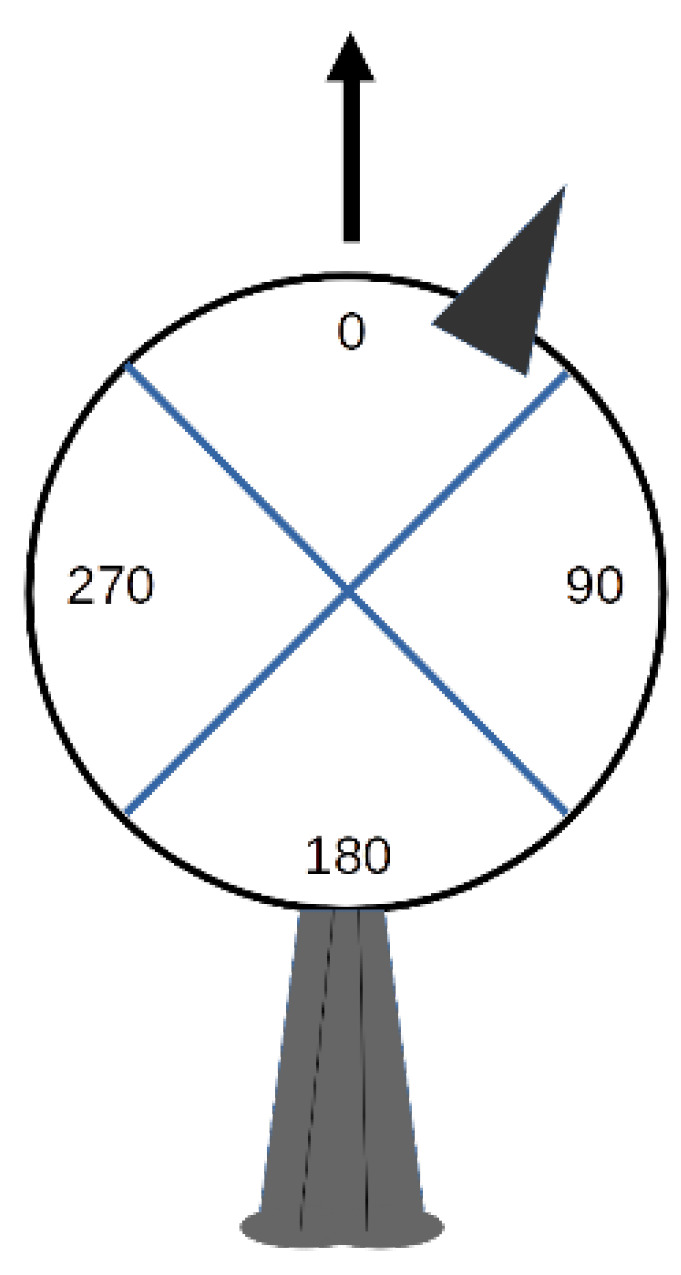
Head orientation of Florida scrub-jay sentinels was determined using the position of the bill (small dark triangle) in one of four possible quadrants from an imaginary circle around the head of the bird. Mid-point angles are shown along with the position of the tail at the bottom. Direction of the long axis of the body is shown with an arrow. Quadrants 1 to 4 correspond to the following mid-point angles: 0, 90, 180, and 270, respectively.

In addition to the duration of looks, the spatial orientation of the head for each look was determined. In our observations, most head movements occurred in the horizontal plane. Therefore, head orientation was obtained by projecting the position of the bill onto an imaginary circle in the horizontal plane centred on the head of the bird (Figure 1).

We then determined in which of four equal segments of 90° (quadrants 1 to 4) the bill was positioned during a look. Viewed from above, the bill at the zero point on the imaginary circle is aligned with the long axis of the body from head to tail and corresponds to the mid-point of quadrant 1. The zero point thus corresponds to the orientation of the head when the head is not turned in any direction. We converted bill positions anywhere in each of the four possible quadrants to angles by using angle values at the mid-point of each quadrant (0°, 90°, 180°, 270°). This scoring system is coarse but realistic given observations in the field at a distance of several meters. Notice that bill position is not necessarily synonymous with gaze direction for animals with eyes set laterally [32]. With these observations, it was possible to determine head orientation (one of four possible angle values) and duration for each look.

With the orientation of the head established for each look, we evaluated return times, namely, how long it took for birds to reorient their heads in quadrant 1 after moving their heads from quadrant 1 to any of the other quadrants. Notice that this is not the equivalent of look duration as several looks can occur within one quadrant before moving on to another quadrant. We chose quadrant 1 as the reference since the head was most frequently oriented in this quadrant (see Section 3).

### 2.3. Statistical Analysis

To analyze look duration, we used a generalized linear mixed model implemented in a Bayesian framework with group ID and individual ID as random effects and group size and focal individual status as fixed effects. The two random effects controlled for multiple focal observations within each group and multiple looks within each focal observation for a given bird, respectively. Group size corresponded to the total number of birds present during a focal observation. Since there were few data for helpers, we classified focal subjects into three possible status categories: juvenile, adult male or adult female.

To proceed with the statistical analysis, we needed to specify the expected distribution of look duration with regular visual monitoring. The duration of a look can be viewed as the outcome of various forces acting independently of one another that either facilitate or hinder threat detection. For instance, look duration might be affected by light level, vegetation cover or the expected number of predators present. If all these forces have an additive effect, the distribution of look duration will follow a normal distribution [33]. The peak in this symmetrical distribution can be viewed as the fixed look duration predicted with regular visual monitoring with noise around it. If the forces, by contrast, have a multiplicative effect, the resulting distribution will be log-normal [33]. This distribution has a peak like the normal distribution (where the preferred look duration of regular visual monitoring is located) but is characterized by a pronounced right skew. If the forces acting on look duration pointed to a high level of risk, for example, an additive process would produce a long look but a multiplicative process would produce an extra long look, which would produce the right skew in the log-normal distribution. For completeness, we also considered distributions that are adapted to individuals that approach surreptitiously like stalkers rather than rely on surprise like aerial predators and neighbours. For such threats, theory predicts that animals should terminate looks at unpredictable times [26,27]. In this case, the distribution of look duration will follow a negative exponential distribution, an ever-decreasing function with a peak at the smallest values. One possible variation for this scenario is that the rate of look interruption is a time-sensitive process rather than the time-insensitive process implicit in the negative exponential distribution [34]. With a rate of interruption that increases with time, for instance, short looks as well as longer looks would become less frequent than expected under the negative exponential distribution, yielding a humped distribution with a right skew. The Weibull family of exponential distributions can be used to model this situation [35].

We fitted four different models to the data with the different types of error distribution suggested above: normal, log-normal, negative exponential, or Weibull. To fit these generalized models, we used Bayesian multilevel models with the *brms* package in R [36]. This Bayesian framework made it possible to include the fixed and random effects described earlier as well as the four different error distributions suggested above. In each model, uninformative priors were used for each parameter. The models ran on four parallel chains of length 2000 with a burn-in of 1000 iterations yielding 4000 values to estimate the posterior distribution for each model parameter. Convergence for the parameter estimates was examined visually by inspecting the Markov chains and by checking whether the convergence statistic (the Gelman-Rubin statistic) was close to 1. This statistic was equal to 1 in all models, indicating convergence.

To determine which of the four possible models was more accurate and could be used to draw inferences, we used cross-validation based on the leave-one-out data splitting scheme [37]. The models were ranked in terms of their expected log pointwise predictive probabilities (ELPD) for new data. The most accurate model is given a score of 0 and the differences in ELPD values relative to this model are calculated for each alternative model (which is similar to rankings with the familiar Akaike criterion). The predictive performance is considered similar if the ELPD difference is 4 or less [37].

We used samples from the posterior distributions generated by the best model to get predicted values in the scale of the data for each status assuming a group size of 4, which was the median group size in the study population. Around the mean of these predicted values, we obtained 95% credible intervals. These intervals give the probability that the effect measured by the parameter falls within the interval given the observed data. When such intervals include the value of 0, it is difficult to reach any definitive conclusion about the absence or presence of effects.

The above procedure established the relative fit of the four models. This does not address the issue of whether the theoretical distribution for the errors used in the best model actually fits the data well. We used the following procedure to assess fit for each focal observation (*n* = 60). The Kolmogorov–Smirnov test (KS) was used to measure the goodness-of-fit to the log-normal distribution, which turned out to be the distribution that provided the best fit (see Section 3). For each focal observation, we fitted a log-normal distribution to the data with the *fitdistrplus* package in R [38]. This procedure produced the KS statistic for the observed distribution of look duration in the focal observation. As the parameters of the log-normal distribution were estimated using the observed data, the *p*-value for the KS statistic is not a proper test of the null hypothesis that the data came from this distribution. To avoid this issue, we used a Monte Carlo approach to generate 1000 synthetic distributions of look duration based on random draws from a log-normal distribution with the estimated parameter values from the focal observation. The sample size in each of the 1000 synthetic distributions was the number of looks in the focal observation. The KS test statistic was calculated for each of the 1000 synthetic samples. We then determined the probability that the KS test statistic from the 1000 synthetic samples was equal or larger than the KS test statistic obtained using the observed data from the focal observation. If this probability was larger than 5%, the log-normal distribution was considered a good fit to the data in the focal observation.

With regular visual monitoring, different areas can be monitored in a systematic fashion. Therefore, sentinels should return their gaze to an already monitored area at regular intervals. By analogy with the distribution of look duration, the distribution of return times under regular visual monitoring should follow either the normal or the log-normal distribution. To analyze the distribution of return times, we used the same generalized linear mixed model approach outlined earlier for look duration. We also fitted the same four different models to the data. Given that the sample size for return times was smaller in each focal observation, we produced the KS test for goodness-of-fit using the whole dataset rather than for each focal observation separately.

As a summary of head orientation during one focal observation, we calculated the mean angle of the orientation weighted by the duration of each look using the *circular* package in R [39]. Assuming the head is turned frequently to each side to achieve broad monitoring, the null hypothesis for the average head orientation is 0°, which means that the head is turned as often and to the same extent on the right side and on the left side of the body. To assess this, we obtained 95% confidence intervals for the average head orientation for each focal observation using a bootstrap procedure based on the von Mises distribution [40]. Biased head orientation (either to the right or the left side) was inferred when the value of 0° was not included in the confidence interval.

## 3. Results

We obtained video recordings from 24 different groups and 45 different individuals (15 adult females, 14 adult males, and 16 juveniles) for a total of 60 focal observations. Ten individuals were observed more than once. Group size ranged from 2 to 6 with a median value of 4 (*n* = 60). Focal observations lasted from 15 to 366 s with a median of 75 s (*n* = 60). Looks lasted from 0.033 to 11.5 s with a median of 0.93 s (*n* = 4582). The median head move duration during a focal observation was either 0.067 or 0.1 s (that is, two or three video frames, *n* = 60).

### 3.1. Look Duration

**Figure 2:**
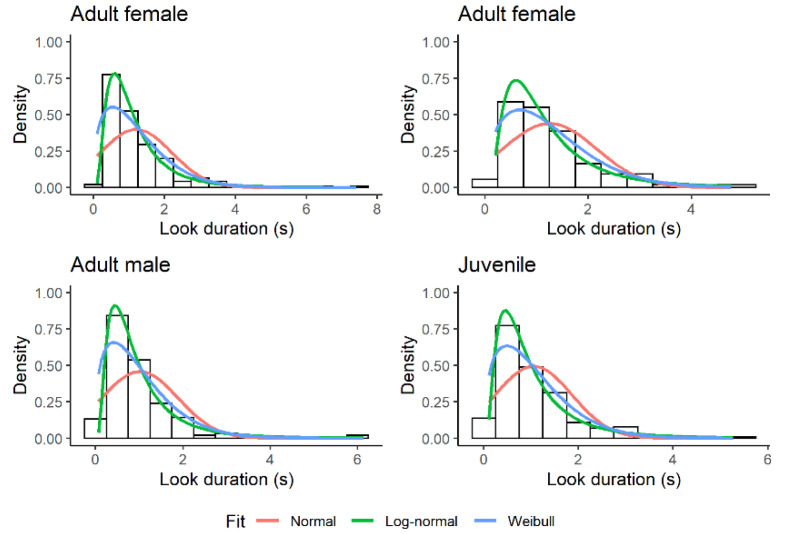
Relative fit of three theoretical distributions to the duration of looks (s) during sentinel bouts in four different Florida scrub-jay sentinels. Fit to the negative exponential distribution is not shown for clarity. The negative exponential distribution is an ever-decreasing function with a peak at the smallest values.

The empirical distribution of look duration was humped and skewed to the right (Figure 2). Of the four models tested, the model with log-normal errors provided the best fit. With respect to this model, the ELPD difference (SE) was 203.9 (32.8) for the Weibull distribution, 576.6 (34.5) for the negative exponential distribution, and 1924.3 (101.1) for the normal distribution. All these ELPD differences were much larger than the threshold of 4. The null hypothesis that the data came from a log-normal distribution was not rejected in any focal observation using the KS test (*n* = 60, *p*-value > 0.24).

**Figure 3:**
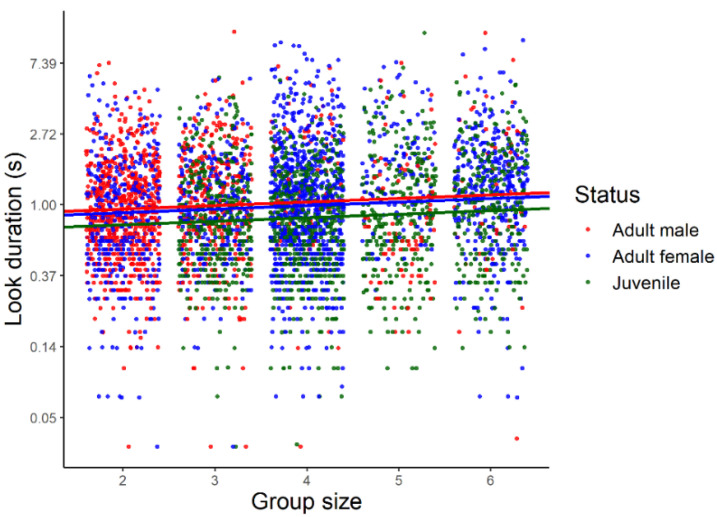
Distribution of look duration (s) during sentinel bouts as a function of group size in three different classes of Florida scrub-jays (juveniles, adult males, and adult females). The duration of looks is in logarithmic scale. Regression lines were obtained from a generalized linear mixed model implemented using a Bayesian framework with log-normal error distribution.

**Figure 4:**
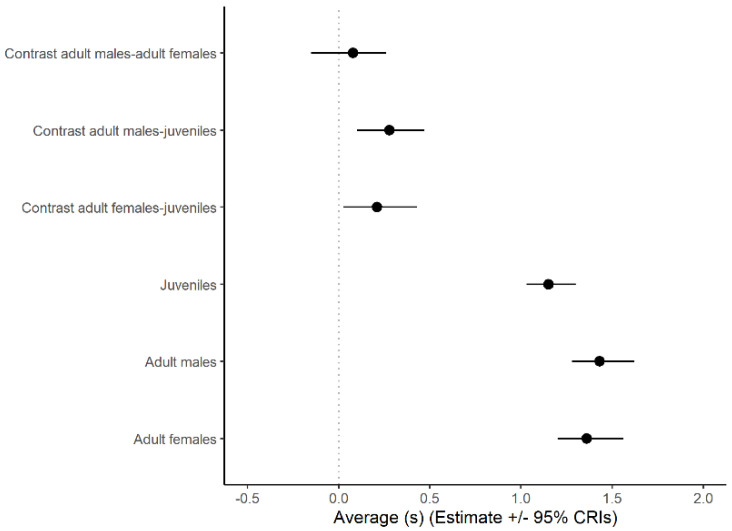
Average look duration (±95% credible intervals) predicted by a generalized linear mixed model implemented using a Bayesian framework with log-normal error distribution for three different classes of Florida scrub-jay sentinels: juveniles, adult males, and adult females. The value of 0 is shown with a vertical line. The average group size was set at four for the calculations.

Drawing inferences from the statistical model based on a log-normal error distribution, the duration of looks increased significantly with group size (β (SE) in log scale: 0.05 (0.02), 95% CrI: 0.01, 0.10, Figure 3). Average look duration also varied with individual status (Figure 4) being larger in adult males and females than in juveniles with no significant difference between adult males and females.

### 3.2. Return Times

**Figure 5:**
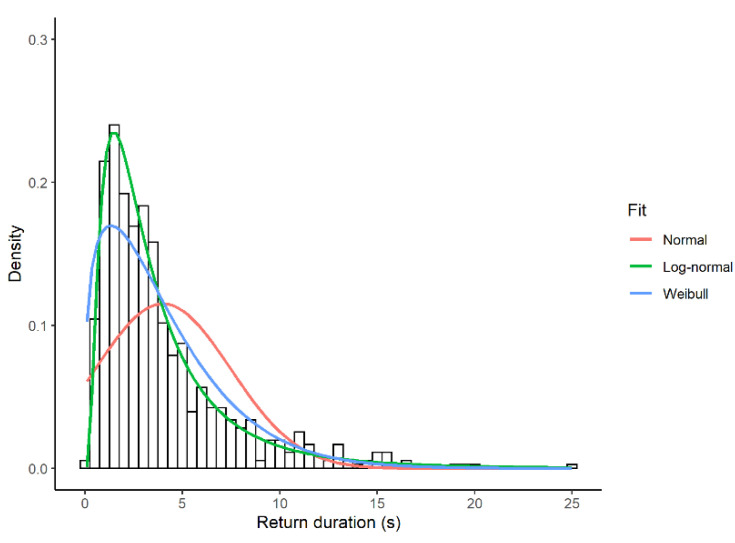
Relative fit of three theoretical distributions to the duration of return times (s) during sentinel bouts in Florida scrub-jay sentinels (*n* = 60). Fit to the negative exponential distribution is not shown for clarity. Return time represents how long it takes for the head to reorient in quadrant 1 after head movements from quadrant 1 to any of the other quadrants. One sentinel bout may contain several return times.

The time needed for the head to return to quadrant 1 after excursions from quadrant 1 to other quadrants varied between 0.1 to 24.9 s, with a median of 2.9 s (*n* = 708). The empirical distribution of return times was humped and skewed to the right (Figure 5). Of the four models tested, the model with log-normal errors provided the best fit. With respect to this model, the ELPD difference (SE) was 33.2 (9.9) for the Weibull distribution, 77 (9.8) for the negative exponential distribution, and 267.2 (24.1) for the normal distribution, values much larger than the threshold of 4. The null hypothesis that the data came from a log-normal distribution was not rejected using the KS test based on all observations (*p*-value = 0.8).

### 3.3. Head Orientation

**Figure 6:**
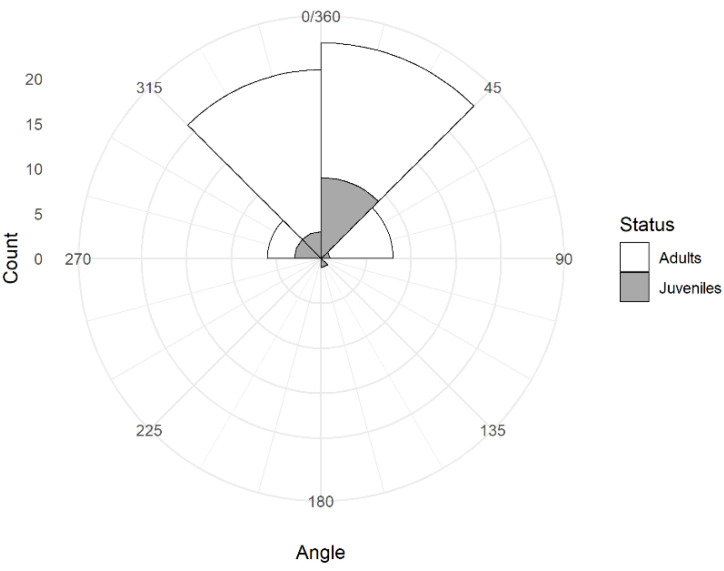
Frequency distribution of average head orientation during sentinel bouts in juvenile and adult Florida scrub-jays (*n* = 60).

Birds moved their heads on average 44 times per min (*n* = 60, range: 21 to 74). Birds moved their heads in all directions but the head was oriented most often in quadrant 1 (mean = 50.7%, range: 18 to 83%) and least often in quadrant 3, the opposite direction (mean = 8.6%, range: 0 to 51%) with intermediate values for quadrant 2 (mean = 18.8%, range: 0 to 61%) and for quadrant 4 (mean = 21.9%, range: 0 to 67%). The distribution of average head orientation among the 60 focal observations was symmetrical around the value of 0° but with substantial inter-individual variation (Figure 6). Biased head orientation (either to the right or the left side of the bird) occurred for all three classes of birds in about 60% of the focal observations, with no obvious preference for the right or the left side of the sentinel (Table 1). The 95% exact confidence intervals around the proportion of birds showing a bias to the right as opposed to the left included the expected value of 0.5 for adult males (proportion = 0.5, 95% CI: 0.26, 0.74), adult females (proportion = 0.53, 95% CI: 0.28, 0.77), and juveniles (proportion = 0.56, 95% CI: 0.21, 0.86).

## 4. Discussion

Various features of sentinel behaviour in Florida scrub-jays support the prediction that, when facing surprise predators and intruders, sentinels should adopt a regular visual monitoring strategy. In particular, sentinels initiated head turns at regular intervals to monitor different areas and also returned their gaze to areas previously monitored at regular intervals. During sentinel bouts, individuals made several head turns, but in many cases head turns were more frequent on one side of the body than the other, which was not predicted by regular visual monitoring. Average look duration during sentinel bouts varied among individuals and among families. In particular, average look duration was shorter in smaller groups and in juveniles.

The log-normal distribution associated with regular visual monitoring provided the best fit to the distribution of look duration and to the distribution of return times, with other theoretical distributions providing relatively poorer fits. In addition, it was not possible to reject the null hypothesis that the data for look duration and for return times came from a log-normal distribution. This strong support for the log-normal distribution indicates that for these two features of sentinel behaviour the various forces shaping look duration acted in a multiplicative rather than in an additive fashion, which explains the right skew in the two empirical distributions. The log-normal distribution also fitted the distribution of look duration when raptors used head turns to search for nearby prey [35]. Right skew suggests that multiplicative processes might be a factor shaping visual search whether for predators or intruders as in Florida scrub-jays or for prey as in raptors.

Biased head orientation was not expected under regular visual monitoring as it implies that some areas are monitored more often than others. We note that not all birds showed side biases and the magnitude of the side biases was often quite small. One possibility to explain biased head orientation is that sentinels in Florida scrub-jays have a favourite eye to carry vigilance, as appears to be the case for other species [41,42,43,44]. However, biases were equally likely on the right or the left side of sentinels, suggesting that it was not always the same eye facing the preferred direction of looking. Biased head orientation might simply mean that the zero point on the imaginary circle around the head of the bird could not always be positioned along the long axis of the body. As sentinel behaviour takes place on top of widely available bushes or small trees, it seems unlikely that sentinels could not change their position to align differently and reduce the need to stretch their necks. One other explanation is that some areas around the sentinels needed more monitoring than others. This appears reasonable at a time of year where territory boundaries are still ill-defined and disputed [20], which means that monitoring neighbours, particularly close to territorial boundaries, might be at a premium. Unfortunately, it was not possible in this study to determine the location of neighbours relative to sentinels to assess whether looks are aimed more often in their direction than predicted. In addition, more data are needed to further assess the extent and magnitude of the side biases.

Average look duration varied systematically from territory to territory. In particular, look duration tended to increase in territories hosting larger groups. In many species, individual investment in sentinel behaviour typically decreases with group size [22,45,46,47]. The results here suggest that group size can affect not only the duration of sentinel bouts but also the way sentinel behaviour is performed. Look duration probably changed with group size in response to variation in perceived predation risk. Theory suggests that predation risk decreases as group size increases. Indeed, the presence of companions can increase the ability to detect threats at the group level and dilute risk among many individuals [48,49]. Lower predation risk in larger groups allows individuals to reduce their investment in vigilance at no increased risk to themselves. Sentinel behaviour in Florida scrub-jays is usually performed alone while other group members forage on the ground [21]. Therefore, sentinels can only rely on themselves to detect threats and will not benefit from mutual warning from other group members. However, risk dilution is still less effective in smaller groups with sentinels. If predation risk is indeed higher in smaller groups, sentinels in such groups could benefit from a decrease in look duration. Shorter looks would allow individuals to turn their heads more frequently thus enhancing visual coverage per unit time. Enhanced visual coverage is at a premium under high risk to reduce the likelihood of failing to detect threats coming from any direction. Support for this idea comes from studies involving different species in which head turns occurred more frequently under high risk [5,7,50]. It is also possible that smaller groups are more at risk from territorial intrusion by neighbours, in which case shorter looks would also be useful to detect intrusions more quickly. More information on how the risk of intrusion varies as a function of group size is needed to assess this idea.

Look duration during sentinel bouts was also shorter in juveniles than in adults. Juvenile Florida scrub-jays typically invest less in sentinel behaviour than adults [22]. The results here suggest that sentinel behaviour itself is also influenced by age. It is perhaps the case that juveniles perceived a higher risk of predation than adults and chose shorter looks to increase visual coverage as noted above in smaller groups. Higher predation risk in juveniles might reflect their relative lack of experience with potential threats. Indeed, inexperience of juveniles is often invoked to explain why vigilance differs as a function of age [51,52]. Sex did not influence either look duration or head orientation at least in adults. In Florida scrub-jays and other species, the length of sentinel bouts tends to be longer in males than females suggesting a role for intra-sexual competition [21,53]. The results here suggest that visual monitoring during sentinel bouts is not sexually selected in this species at this time of year.

We consider head movements and subsequent looks as a primarily visual search task allowing sentinels to monitor areas around them for threats. Looks could also be used to track objects of interests, such as predators or neighbours, once identified through visual search [29]. In this study, visual tracking of predators appears unlikely since we did not detect alarm calls associated with aerial predators during focal observations. We minimized visual tracking of close intruders by avoiding observations when groups interacted. Nevertheless, visual tracking of distant neighbours is a possibility that cannot be excluded since we did not know the position of neighbours relative to sentinels. Future research is needed to establish how often distant neighbours are present during sentinel bouts and whether looks vary in duration when they are.

The approach used here, which combines quantitative measurements, such as look duration and head orientation and the use of theoretical distributions expected under different threat scenarios, could be used in other species with sentinel behaviour to determine the robustness of the findings. In addition, this approach could be used in different contexts such as when foraging bouts are interrupted by vigilance. It is perhaps the case that regular visual monitoring applies well to many situations involving visual search be it for predators, intruders or prey. Such studies will be useful to determine how visual monitoring strategies reflect the type of threats faced by animals.

## 5. Conclusions

Florida scrub-jays during sentinel bouts adopted a regular pattern of vigilance predicted when predators and intruders rely on surprise to approach. Biased head orientation in sentinel bouts might be related to the need to monitor particular neighbours, but this requires more work. Looks were shorter in smaller groups and in juveniles presumably to increase visual coverage in more threatening situations. These findings highlight how visual monitoring strategies during vigilance are tailored to perceived threats.

## Figures and Tables

**Table 1:** Statistically significant departures from the null expectation that average head orientation is equal to 0° for each status of Florida scrub-jay sentinels (*n* = 60).

Status	Bias to the Left	No Bias	Bias to the Right	*n*
Adult females	6	7	6	19
Adult males	8	7	9	24
Juveniles	4	8	5	17

## Data Availability

The data are available from the authors.

## References

[1] Beauchamp G. (2015). Animal Vigilance: Monitoring Predators and Competitors.

[2] Lima S.L. (1987). Vigilance while feeding and its relation to the risk of predation. J. Theor. Biol..

[3] McNamara J.M., Houston A.I. (1992). Evolutionarily stable levels of vigilance as a function of group size. Anim. Behav..

[4] Sirot E. (2019). Adjustments in compound defensive strategies in response to variation in predation risk. Anim. Behav..

[5] Jones K.A., Krebs J.R., Whittingham M.J. (2007). Vigilance in the third dimension: Head movement not scan duration varies in response to different predator models. Anim. Behav..

[6] Fernández-Juricic E. (2012). Sensory basis of vigilance behavior in birds: Synthesis and future prospects. Behav. Proc..

[7] Fernández-Juricic E., Beauchamp G., Treminio R., Hoover M. (2011). Making heads turn: Association between head movements during vigilance and perceived predation risk in brown-headed cowbird flocks. Anim. Behav..

[8] Bekoff M. (1995). Vigilance, flock size, and flock geometry—Information gathering by western evening grosbeaks (Aves, Fringillidae). Ethology.

[9] Lazarus J. (1979). Flock size and behaviour in captive red-billed weaverbirds (*Quelea quelea)*: Implications for social facilitation and the functions of flocking. Behaviour.

[10] Cézilly F., Brun B. (1989). Surveillance et picorage chez la tourterelle rieuse, *Streptopelia risoria*: Effets de la présence d’un congénère et de la dispersion des graines. Behaviour.

[11] Favreau F.-R., Goldizen A.W., Pays O. (2010). Interactions among social monitoring, anti-predator vigilance and group size in eastern grey kangaroos. Proc. R. Soc. Lond. B Biol. Sci..

[12] Hirsch B.T. (2002). Social monitoring and vigilance behavior in brown capuchin monkeys (*Cebus apella*). Behav. Ecol. Sociobiol..

[13] Beauchamp G. (2001). Should vigilance always decrease with group size?. Behav. Ecol. Sociobiol..

[14] Coolen I., Giraldeau L.A., Lavoie M. (2001). Head position as an indicator of producer and scrounger tactics in a ground-feeding bird. Anim. Behav..

[15] Li M.F., Arseneau-Robar T.J.M., Smeltzer E.A., Teichroeb J.A. (2021). Be early or be tolerated: Vervet monkey, Chlorocebus pygerythrus, foraging strategies in a dispersed resource. Anim. Behav..

[16] Dawkins M.S. (2002). What are birds looking at? Head movements and eye use in chickens. Anim. Behav..

[17] Bednekoff P.A. (2015). Sentinel behavior: A review and prospectus. Adv. Study Behav..

[18] Bell M.B.V., Radford A.N., Rose R., Wade H.M., Ridley A.R. (2009). The value of constant surveillance in a risky environment. Proc. R. Soc. Lond. B Biol. Sci..

[19] Bednekoff P.A. (1997). Mutualism among safe, selfish sentinels: A dynamic game. Am. Nat..

[20] Woolfenden G.E., Fitzpartrick J.W. (1984). The Florida Scrub-Jay: Demography of a Cooperative-Breeding Bird.

[21] McGowan K.J., Woolfenden G.E. (1989). A sentinel system in the Florida scrub jay. Anim. Behav..

[22] Hailman J.P., McGowan K.J., Woolfenden G.E. (1994). Role of helpers in the sentinel behaviour of the Florida scrub jay (*Aphelocoma c. coerulescens*). Ethology.

[23] Bednekoff P.A., Woolfenden G.E. (2003). Florida scrub-jays (*Aphelocoma coerulescens*) are sentinels more when well-fed (even with no kin nearby). Ethology.

[24] Bednekoff P.A., Woolfenden G.E. (2006). Florida Scrub-Jays compensate for the sentinel behavior of flockmates. Ethology.

[25] Woolfenden G.E., Fitzpatrick J.W., Poole A.F., Gill F.B. (2020). Florida Scrub-Jay (*Aphelocoma coerulescens*). Birds of the World.

[26] Scannell J., Roberts G., Lazarus J. (2001). Prey scan at random to evade observant predators. Proc. R. Soc. Lond. B Biol. Sci..

[27] Bednekoff P.A., Lima S.L. (2002). Why are scanning patterns so variable? An overlooked question in the study of anti-predator vigilance. J. Avian Biol..

[28] Fernández-Juricic E., O’Rourke C., Pitlik T. (2010). Visual coverage and scanning behavior in two corvid species: American crow and Western scrub jay. J. Comp. Physiol. A-Neuroethol. Sens. Neural Behav. Physiol..

[29] Land M.F. (1999). Motion and vision: Why animals move their eyes. J. Comp. Physiol. A-Neuroethol Sens. Neural Behav. Physiol..

[30] Beauchamp G. (2017). Half-blind to the risk of predation. Front Ecol. Evol..

[31] Yorzinski J.L. (2016). Eye blinking in an avian species is associated with gaze shifts. Sci. Rep..

[32] Dawkins M.S. (1995). How do hens view other hens? The use of lateral and binocular fields in social recognition. Behaviour.

[33] Limpert E., Stahel W.A., Abbt M. (2001). Log-normal distributions across the sciences: Keys and clues. Bioscience.

[34] Lendrem D.W., Stretch D., Metcalfe N.B., Jones P. (1986). Scanning for predators in the purple sandpiper: A time-dependent or time-independent process?. Anim. Behav..

[35] Ochs M.F., Zamani M., Gomes G.M.R., de Oliveira Neto R.C., Kane S.A. (2016). Sneak peek: Raptors search for prey using stochastic head turns. AUK.

[36] Bürkner P.-C. (2017). brms: An R Package for Bayesian Multilevel Models Using Stan. J. Stat. Softw..

[37] Vehtari A., Gelman A., Gabry J. (2017). Practical Bayesian model evaluation using leave-one-out cross-validation and WAIC. Stat. Comput..

[38] Delignette-Muller M.L., Dutang C. (2015). fitdistrplus: An R Package for Fitting Distributions. J. Stat. Softw..

[39] Agostinelli C., Lund U. 2022 R Package ‘Circular’: Circular Statistics, Version 0.4-95. https://r-forge.r-project.org/projects/circular/.

[40] Ruxton G.D. (2017). Testing for departure from uniformity and estimating mean direction for circular data. Biol. Lett..

[41] Franklin W.E., Lima S.L. (2001). Laterality in avian vigilance: Do sparrows have a favourite eye?. Anim. Behav..

[42] Randler C. (2005). Eye preference for vigilance during feeding in coot *Fulica atra*, and geese *Anser anser* and *Anser cygnoides*. Laterality.

[43] Fourie B., Berezina E., Giljov A., Karenina K. (2021). Visual lateralization in artiodactyls: A brief summary of research and new evidence on saiga antelope. Laterality.

[44] Beauchamp G. (2013). Foraging success in a wild species of bird varies depending on which eye is used for anti-predator vigilance. Laterality.

[45] Rasa O.A.E. (1989). The costs and effectiveness of vigilance behaviour in the dwarf mongoose: Implications for fitness and optimal group size. Ethol. Ecol. Evol..

[46] Clutton-Brock T.H., O’Riain M.J., Brotherton P.N.M., Gaynor D., Kansky R., Griffin A.S., Manser M. (1999). Selfish sentinels in cooperative mammals. Science.

[47] Ridley A.R., Raihani N.J. (2007). Facultative response to a kleptoparasite by the cooperatively breeding pied babbler. Behav. Ecol..

[48] Pulliam H.R. (1973). On the advantages of flocking. J. Theor. Biol..

[49] Bertram B.C.R., Krebs J.R., Davies N.B. (1978). Living in groups: Predator and prey. Behavioural Ecology.

[50] Moore B.A., Doppler M., Young J.E., Fernández-Juricic E. (2013). Interspecific differences in the visual system and scanning behavior of three forest passerines that form heterospecific flocks. J. Comp. Physiol. A-Neuroethol. Sens. Neural. Behav. Physiol..

[51] Griffin A.S., Evans C.S., Blumstein D.T. (2001). Learning specificity in acquired predator recognition. Anim. Behav..

[52] Stankowich T., Blumstein D.T. (2005). Fear in animals: A meta-analysis and review of fear assessment. Proc. R. Soc. Lond. B Biol. Sci..

[53] Walker L.A., York J.E., Young A.J. (2016). Sexually selected sentinels? Evidence of a role for intrasexual competition in sentinel behavior. Behav. Ecol..

